# Enhanced Thermoelectric Properties of Te Doped Polycrystalline Sn_0.94_Pb_0.01_Se

**DOI:** 10.3390/nano12091575

**Published:** 2022-05-06

**Authors:** Fujin Li, Lin Bo, Ruipeng Zhang, Sida Liu, Junliang Zhu, Min Zuo, Degang Zhao

**Affiliations:** 1 School of Materials Science and Engineering, University of Jinan, Jinan 250022, China; happytogetherl@163.com (F.L.); bling828@163.com (L.B.); zhangrp163@163.com (R.Z.); itachisongja@gmail.com (J.Z.); mse_zuom@ujn.edu.cn (M.Z.); 2 Institute for Advanced Technology, Shandong University, Jinan 250012, China; sidaliu@sdu.edu.cn

**Keywords:** SnSe, nanoparticles, *zT*, thermoelectric, TEM

## Abstract

Thermoelectric materials can directly convert heat and electricity, which is a kind of promising energy material. In view of cost and mechanical properties, polycrystalline SnSe material with high *zT* value is greatly desired. In this study, polycrystalline Sn_0.94_Pb_0.01_Se_1-*x*_Te*_x_* samples were prepared by the vacuum melting–hot pressing sintering method. Sn vacancies, Pb and Te atoms were simultaneously introduced into the polycrystalline SnSe. The power factor of Sn_0.94_Pb_0.01_Se_1-*x*_Te*_x_* samples was decreased, which could be attributed to the generation of *n*-type semiconductor SnSe_2_. In addition, the phonons were strongly scattered by point defects and dislocations, which led to the decrease of thermal conductivity—from 0.43 Wm^−1^K^−1^ to 0.29 Wm^−1^K^−1^ at 750 K. Finally, the polycrystalline Sn_0.94_Pb_0.01_Se_0.96_Te_0.04_ sample achieved the maximum *zT* value of 0.60 at 750 K.

## 1. Introduction

Thermoelectric material, as a kind of sustainable material that can directly convert heat and electricity, has great application prospects in the power generation and semiconductor cooling [1]. The conversion efficiency of thermoelectric material is dependent on the dimensionless figure of merit

$$zT=\frac{\sigma S^{2}}{\kappa_{L}+\kappa_{e}}T$$

where *S* is Seebeck coefficient, *σ* is electrical conductivity, *σS*^2^ is power factor (*PF*), *T* is operating temperature and *κ_L_* and *κ_e_* are lattice thermal conductivity and electronic thermal conductivity, respectively [2]. To obtain a high *zT*, thermoelectric material should have a high power factor and low thermal conductivity [3]. However, the coupling between *σ*, *S* and *κ_e_* in the thermoelectric materials makes it difficult to obtain high *zT*. The decoupling of the three parameters has become an important trend to improve the performance of thermoelectric materials.

Single crystal SnSe with a layered structure has been widely studied due to its excellent thermoelectric properties [4,5,6,7,8,9,10]. However, due to the poor mechanical properties and the long production cycle, practical application of single crystal SnSe has been limited. Therefore, polycrystalline SnSe with good mechanical properties and a simple production process has received considerable attention. The low electrical properties and high thermal conductivity are the major obstacles to the practical application of polycrystalline SnSe. At present, the thermoelectric properties of polycrystalline SnSe can be optimized by enhancing the power factor through band engineering (resonance energy levels and band convergence) [11,12,13] and the energy filtering effect [14], or by reducing the lattice thermal conductivity through nanostructure [15,16,17,18] and full-scale hierarchy [19,20]. In previous studies, the doping of Ag [21], Cu [22], alkaline ions [23], In [24], S [25], Zn [26] and other chemical elements proved that doping is an effective approach to optimize the thermoelectric properties of polycrystalline SnSe. In addition, by introducing Sn vacancy in SnSe, the Fermi level can be deepened into the valence band, and the energy band near the Fermi level can be smoothened. Therefore, the Fermi level has a larger energy state density, and the carrier concentration can be increased. Moreover, the appearance of an Sn vacancy can destroy the translational symmetry of SnSe, which could reduce the lattice thermal conductivity with the increase of phonon scattering centers [15]. Recently, co-doping was proven to be a reasonable method to enhance the thermoelectric properties of polycrystalline SnSe. Chang et al. achieved high *zT* value of ~1.2 at 773 K in *n*-type Br and Pb co-doped polycrystalline SnSe [27]. Lee et al. synthesized Na and Pb co-doped polycrystalline SnSe, which obtained a *zT* value of ~1.2 at 773 K [28]. Tang et al. obtained a high *zT* value of 2.2 via phase separation and nanostructuring strategies in Pb and Zn co-doped polycrystalline SnSe [29]. These results indicated that co-doping has great benefits for improving the thermoelectric properties of polycrystalline SnSe. Therefore, Sn vacancies and Pb and Te co-doping were employed to enhance thermoelectric properties of polycrystalline SnSe.

In this study, the *p*-type polycrystalline Sn_0.94_Pb_0.01_Se_1-*x*_Te*_x_* (SPST) samples were prepared by the vacuum melting–hot pressing sintering method. The influences of Te doping on the microstructure and thermoelectric properties of SPST samples were investigated. The nano-PbTe formed in SPST samples resulted in phonon scattering, which effectively reduced the thermal conductivity and enhanced the thermoelectric properties. It is expected to provide a useful guide and reference for the development of polycrystalline SnSe thermoelectric material.

## 2. Materials and Methods

High-purity Sn (99.99%, Aladdin), Pb (99.99%, Aladdin), Se (99.99%, Aladdin) and Te (99.99%, Aladdin) powders were weighed according to the atomic ratio of Sn_0.94_Pb_0.01_Se_1-*x*_Te*_x_* (*x* = 0, 0.01, 0.02, 0.03, 0.04). The raw materials were loaded into a graphite crucible and then sealed in quartz ampoules under vacuum. The ampoules were slowly heated up to 1223 K over 9.5 h and then maintained at this temperature for 6 h; they were then cooled down to 873 K over 3.5 h and annealed at 873 K for 72 h. The annealed ingots were ground into powder with an agate mortar and then ball milled for 20 h. Finally, the bulk SPST samples (wafer, φ = 12 mm, thickness = 1.0–1.2 mm) were sintered at 773 K for 30 min under a pressure of 50 MPa by rapid hot-pressing sintering.

Powder X-ray diffraction (XRD) analysis was performed on a Hitachi X-ray diffractometer (Cu Kα radiation, Tokyo, Japan). Transmission electron microscopy (TEM) analyses, including TEM imaging and high resolution transmission electron microscope (HRTEM) imaging, were performed on a probe-corrected microscope FEI Talos-F200S (ThermoFisher Scientific, Beijing, China) at 200 KV. The samples for TEM were prepared by focused ion beam (FIB) using the lift-out method from the bulk samples. The electrical conductivity and Seebeck coefficient of all samples were measured by commercial equipment (ZEM-3, ULVAC-RIKO, Yokohama, Japan). All measurements were carried out in the temperature range of 300–750 K. The thermal conductivity of all samples was calculated according to the formula *κ* = *ρ*D*C_p_* (*ρ* is the density of the material, D is the thermal diffusion coefficient of the material, and *C_p_* is the specific heat capacity of material). The density of all samples was measured by the Archimedes drainage method. The specific heat capacity (*C_p_*) was taken from reference [4]. The thermal diffusivity of SPST samples was measured by the laser flash method (Netzsch LFA-457, Selb, Germany) in a flowing Ar atmosphere. The Hall coefficient *R_H_* of the sample was measured using the van der Pauw technique under a reversible magnetic field of 0.8 T at room temperature. The diameter of samples was about 12 mm, and the thickness was 1.0–1.2 mm. The carrier concentration (*n_H_*) was calculated by *n_H_* = 1/(*eR_H_*), and carrier mobility (*μ_H_*) was calculated according to *μ_H_* = *σ⋅R_H_*, where *e* is the unit charge, and *σ* is the electrical conductivity. In addition, *PF* was calculated by *PF* = *σS^2^*, and *zT* was calculated according to *zT* = (*σS*^2^)/(*κ_L_* + *κ_e_*) *T*. The test uncertainty of electrical conductivity, Seebeck coefficient and thermal conductivity was about 5%. Error bars are not shown in the figures in order to increase the readability of data curves.

## 3. Results and Discussion

**Figure 1:**
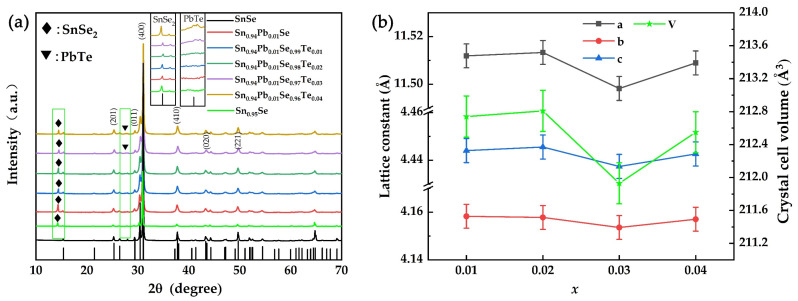
(**a**) X-ray diffraction pattern of SPST samples. (**b**) Lattice constant and crystal cell volume of SPST samples.

The X-ray diffraction (XRD) patterns of SPST samples and pristine SnSe are shown in Figure 1a. The Pnma orthorhombic phase of SnSe (PDF#48-1224) was well indexed. In addition, the *n*-type SnSe_2_ phase was found in SPST samples, as indicated by the diamond in Figure 1a. It can be speculated that the formation energy of Sn vacancy decreased during the annealing process, which led to the appearance of SnSe_2_. With the increasing of Te content, the PbTe phase could also be observed in the XRD pattern, as indicated by the inverted triangle in Figure 1a. The precipitation of PbTe was possibly caused by the reduced solubility of Pb in SnSe due to the excessive Te. The precipitation of PbTe was indirectly proven by the lattice constant and crystal cell volume of SPST samples. Figure 1b shows the lattice constant and crystal cell volume. The error range of the lattice constants of doped SnSe were similar to those in previous reports [22,24]. With the increase of Te content, the lattice constant and crystal cell volume of SPST samples increased when *x* ≤ 0.02. However, the lattice constant and crystal cell volume of SPST samples were decreased when *x* = 0.03, which indicated the decrease of Pb with large radius in SPST samples, and the precipitated Pb and Te formed the precipitation of PbTe. The subsequent increases of the lattice constant and crystal cell volume were due to increases of Te in SPST samples.

Table 1 lists the density and relative density of all samples at room temperature. It can be seen that the relative density of all samples exceeded 95%. Therefore, the effect of porosity on the TE properties of all samples was negligible.

**Figure 2:**
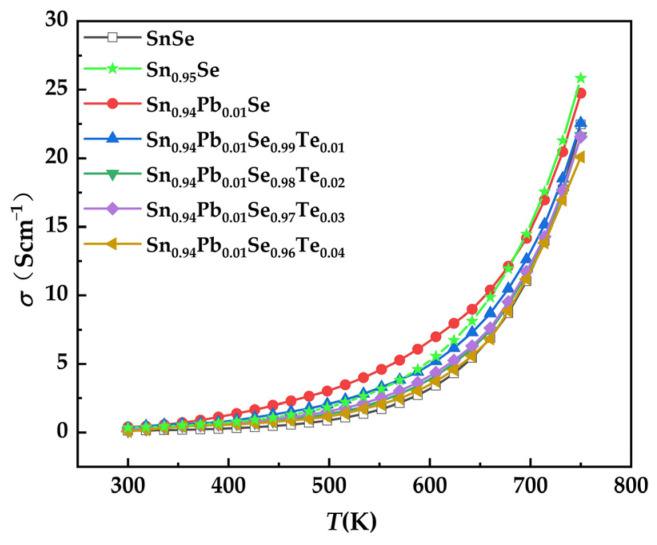
Electrical conductivity of SPST samples as a function of temperature.

Figure 2 shows the electrical conductivity of SPST samples as a function of temperature. The electrical conductivity of all samples increased as the temperature rose, exhibiting the typical semiconductor characteristics. The electrical conductivity of SPST samples decreased as the content of Te-doping increased. Furthermore, the thermal excitation of carriers caused a dramatic increase in electrical conductivity at ~650 K.

**Figure 3:**
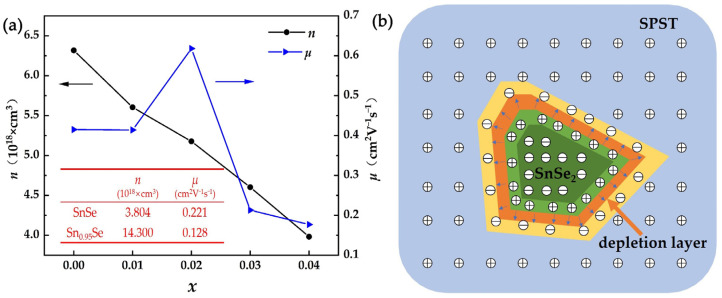
(**a**) The carrier concentration and mobility of all samples at room temperature, (**b**) depletion layer between SnSe and SnSe_2_.

The variation of electrical conductivity at room temperature can be understood with insight into carrier concentration and carrier mobility in SPST samples. As shown in Figure 3a, compared with the carrier concentration of SnSe, the carrier concentration of Sn_0.95_Se and SPST samples increased due to the introduction of Sn vacancies [15]. In addition, the carrier concentration of SPST samples were much lower than that of Sn_0.95_Se. It could be speculated that the Pb substitution on Sn sites reduced the intrinsic Sn vacancies, which were the major source of charge carriers in *p*-type SnSe [23]. With the amount of Te-doping increasing, the carrier concentration of SPST samples decreased from 6.317 × 10^18^ cm^3^ for Sn_0.94_Pb_0.01_Se to 3.983 × 10^18^ cm^3^ for Sn_0.94_Pb_0.01_Se_0.96_Te_0.04_, which was due to the enlarged band gap. The enlarged band gap led to the obstruction of electrons that transit from the valence band to the conduction band, which resulted in the decrease of carrier concentration. The energy gap *E_g_* can be estimated from the equation *E_g_* ≈ 2*e* |*S_max_*|*T_Smax_*, where *e* is the electron charge [30]. The calculated energy gap values *E_g_* are shown in Table 2. Moreover, holes and electrons diffused and recombined at the interface between *n*-type SnSe_2_ and *p*-type SPST semiconductors under the concentration difference, which formed the depletion layer, as shown in Figure 3b. Correspondingly, the opposite charges appeared at the original position, which led to the formation of a built-in electric field at the interface region. The direction of the electric field is shown by the blue arrow in Figure 3b. The movement of lower energy carriers were limited by the built-in electric field, which decreased the carrier concentration and carrier mobility. Therefore, the carrier concentration was further reduced by the built-in electric field between the *p*-type SPST and *n*-type SnSe_2_.

The carrier mobility of Sn_0.95_Se was lower than that of SPST samples, as the electronegativity difference between Pb (2.33) and Se (2.55) was lower than that between Sn (1.96) and Se (2.55). Similarly, the electronegativity difference between Sn (1.96) and Te (2.1) was also lower than that between Sn (1.96) and Se (2.55). This indicated that the bonding of Pb–Se [31] and Sn–Te [32] had a covalent character, which resulted in the weakened electron localization and increased carrier mobility [33]. With the increase of Te content, the carrier mobility of SPST samples increased when *x* ≤ 0.02. However, the carrier mobility of SPST samples significantly decreased when *x* > 0.02, which was caused by the intensification of electron localization in SPST due to the precipitation of PbTe. Furthermore, the carrier mobility was reduced, caused by the depletion layer between the SPST and SnSe_2_ in SPST samples.

**Figure 4:**
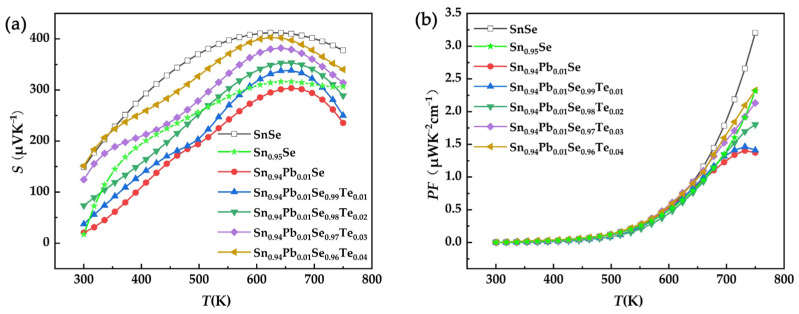
(**a**) Seebeck coefficient and (**b**) power factor of SPST samples as a function of temperature.

Figure 4a shows the temperature dependence of the Seebeck coefficient for SPST samples. The *S* of all samples presented a positive value over the whole temperature range, indicating all SPST samples were *p*-type semiconductors. With increases in temperature, the *S* reached a maximum value at about ~650 K, which was due to the thermal excitation of minority carriers (bipolar effect), and the same trends were also reported in previous literatures [29,34,35,36]. With the increase of Te content, the *S* increased, which is likely related to the decreased *n*. Figure 4b shows the power factor of SPST samples as a function of temperature. Due to the deterioration of the Seebeck coefficient, the power factor of SPST samples was slightly degraded. The maximum power factor of the Sn_0.94_Pb_0.01_Se_0.96_Te_0.04_ sample was only 2.33 μWK^−2^ cm^−1^ at 750 K.

**Figure 5:**
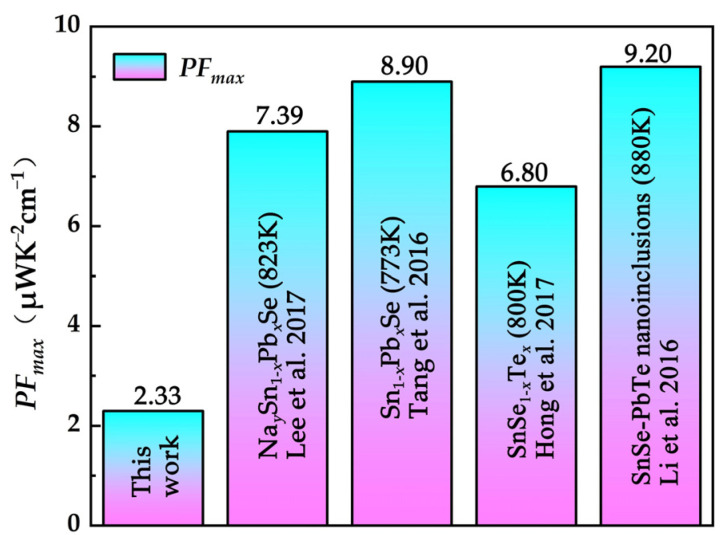
Comparison of *PF_max_* of doped SnSe.

As shown in Figure 5, compared with the SnSe_1-*x*_Te*_x_* samples [37], Sn_1-*x*_Pb*_x_*Se samples [28,31] and SnSe-based composites with PbTe nanoinclusions [38], the *PF_max_* of SPST samples was lowest, which was due to the decreased electrical conductivity and *S*.

**Figure 6:**
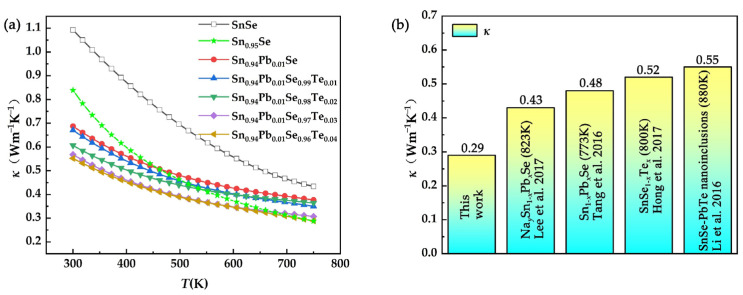
(**a**) Total thermal conductivity of SPST samples as a function of temperature. (**b**) Comparison of total thermal conductivity of doped SnSe.

**Figure 7:**
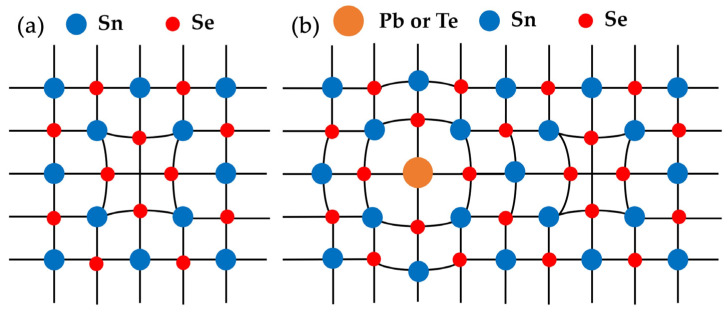
The schematic diagram of lattice distortion. (**a**) Sn_0.95_Se and (**b**) SPST samples.

Figure 6a shows the temperature dependence of thermal conductivity for SPST samples. Compared with pristine SnSe, the *κ* of Sn_0.95_Se and SPST samples decreased significantly. The *κ* of Sn_0.95_Se was higher than that of SPST samples at low temperature, which was mainly due to the fluctuations of the mass field and stress field in the SPST samples. Inversely, with increasing temperature, the *κ* of Sn_0.95_Se decreased compared to that of SPST samples. In the Pnma phase of SnSe, the (100) plane with retractable characteristics contributed to the low *κ*, which was called the soft lattice [35]. According to a previous report [35], the soft lattice was rigidified by stronger atomic bonds, which could lead to a slight improvement in the phonon conduction. In the Sn_0.95_Se sample, the Sn vacancy contributed to long or missing interatomic linkages [39], leading to weaker atomic bonds, which further enhanced soft lattice, as shown in Figure 7a. However, when Sn vacancy coexisted with Pb/Te, Pb or Te with larger atomic radius, the bond length was compressed between atoms, and a stronger atomic bond than that of Sn_0.95_Se formed, as shown in Figure 7b. The thermal conductivity was mainly dominated by phonon–phonon scattering at high temperature, resulting in the higher thermal conductivity of SPST samples than that of Sn_0.95_Se. The lowest *κ* of 0.29 Wm^−1^K^−1^ in the Sn_0.94_Pb_0.01_Se_0.96_Te_0.04_ sample was obtained at 750 K, which was slightly difference than that of Sn_0.95_Se. As shown in Figure 6b, compared with the SnSe_1-*x*_Te*_x_* samples [37], Sn_1-*x*_Pb*_x_*Se samples [28,31] and SnSe-based composites with PbTe nanoinclusions [38], the total thermal conductivity of SPST samples was lowest, which indicated that the thermal conductivity of SPST samples was effectively decreased by multi-atomic doping. Moreover, with the Te content increasing, the *κ* of SPST samples decreased, which indicated that the heterogeneous atoms play an important role in optimizing the thermal conductivity. Due to the low electrical conductivity of all samples, the electron thermal conductivity was negligible.

**Figure 8:**
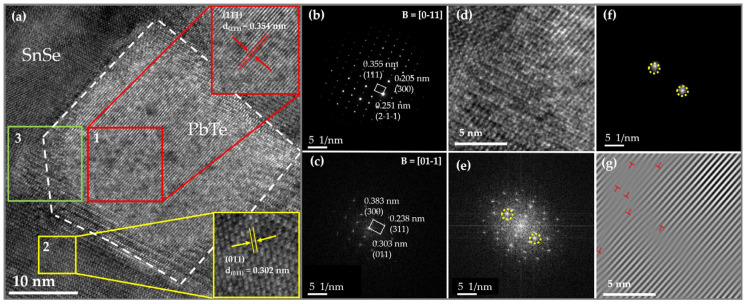
(**a**) High-resolution transmission electron microscopy (HRTEM) images of the Sn_0.94_Pb_0.01_Se_0.96_Te_0.04_ sample. (**b**) The Fast Fourier Transform (FFT) patterns of area 1 in (**a**). (**c**) FFT patterns of area 2 in (**a**). (**d**) Area 3 in (**a**). (**e**) FFT patterns of (**d**). (**f**) The filtered FFT by applying the mask to a specific diffraction spot in (**e**). (**g**) The dislocation maps obtained by local iFFT of (**f**).

To further investigate the microstructures of SPST samples, TEM was employed for the Sn_0.94_Pb_0.01_Se_0.96_Te_0.04_ sample. As shown in Figure 8a, some nanoparticles with the size of ~35 nm were observed in the Sn_0.94_Pb_0.01_Se_0.96_Te_0.04_ sample. The corresponding FFT patterns for nanoparticles and matrix are shown in Figure 8b,c, respectively. The zone axis of the FFT pattern for the nanoparticle was [0−11], and the zone axis of FFT for the matrix was [01−1]. The distances from the three diffraction spots to the central transmission point were 0.251 nm, 0.355 nm and 0.205 nm, respectively, which corresponded to the (2−1−1), (111) and (300) planes of the cubic PbTe phase, respectively. The formation of PbTe nanoparticles was attributed to the excess Te, which influenced the solubility of Pb in SnSe. Similarly, the minimum interplanar spacings of the FFT pattern for the matrix in different directions were 0.303 nm, 0.383 nm and 0.238 nm, respectively, which matched well with (011), (300) and (311) planes of SnSe, respectively. As shown in area 3 of Figure 8a, PbTe and the matrix exhibited a slightly lattice mismatch, which could lead to the formation of dislocations. Therefore, iFFT was performed in area 3 of Figure 8a. Many dense dislocations could be found, as shown in Figure 8g. The dense dislocations increased the phonon scattering center, which strongly scattered the phonons. It is clear that the dense dislocations and nanoscale PbTe phase increased the anharmonicity of the lattice, which could strongly scatter mid-frequency phonons and significantly reduce the lattice thermal conductivity.

**Figure 9:**
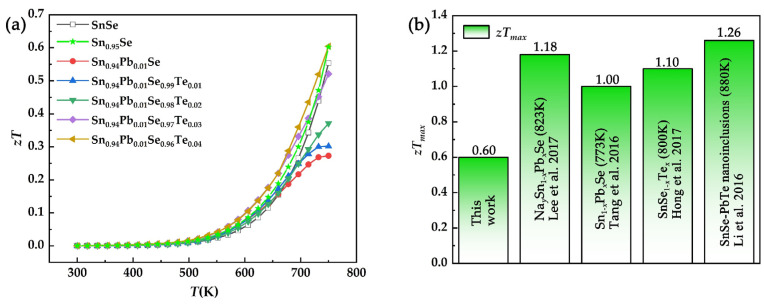
(**a**) *zT* of SPST samples as a function of temperature. (**b**) Comparison of *zT_max_* of doped SnSe.

Figure 9a shows the figure of merit of SPST samples as a function of temperature. Due to the extremely low thermal conductivity, the Sn_0.94_Pb_0.01_Se_0.96_Te_0.04_ obtained a maximum *zT* of 0.60 at 750 K. However, as shown in Figure 9b, compared with the SnSe_1-*x*_Te*_x_* samples [37], Sn_1-*x*_Pb*_x_*Se samples [28,31] and SnSe-based composites with PbTe nanoinclusions [38], the *zT_max_* of SPST samples was lowest, which was attributed to the deterioration of electrical properties. This result indicated that *zT* of SPST was greatly weakened due to the appearance of *n*-type SnSe_2_.

At 750 K, the numerical values of the various parameters for all the materials tested were compiled, as shown in Table 3, which indicated the increase of *zT* was mainly due to the decrease of *κ* caused by the heterogeneous atoms. Furthermore, the thermoelectric performance of SPST samples was greatly deteriorated due to the presence of SnSe_2_, and so the elimination of SnSe_2_ was necessary.

## 4. Conclusions

Polycrystalline Sn_0.94_Pb_0.01_Se_1-x_Te_x_ (*x* = 0~0.04) samples were prepared by the vacuum melting–hot pressing sintering method. Due to the decrease of Sn vacancy formation energy, the SnSe_2_ formed in polycrystalline SnSe samples with Sn vacancies, and nano-PbTe appeared in SPST samples caused by excessive Te. The carrier concentration was increased by Sn vacancies. The carrier mobility was effectively increased due to the low electronegativity difference by Pb and Te substitution. However, due to formation of an *n*-type SnSe_2_ phase, the electrical properties of polycrystalline Sn_0.94_Pb_0.01_Se_1-*x*_Te*_x_* samples were deteriorated, and a maximum power factor of 2.33 μWK^−2^ cm^−1^ was obtained at 750 K. Moreover, the heterogeneous atoms led to an incredible decrease in thermal conductivity. The phonons were strongly scattered due to the point defects formed by heterogeneous atoms, nano-PbTe and dense dislocations, and the lowest thermal conductivity of 0.29 Wm^−1^K^−1^ was obtained for Sn_0.94_Pb_0.01_Se_0.96_Te_0.0__4_ at 750 K, which was 33% lower than that of pristine SnSe. On the whole, the thermoelectric performance of polycrystalline SnSe could be effectively increased by the doping of Sn vacancies, Pb and Te. The highest *zT* value of 0.60 was obtained for the Sn_0.94_Pb_0.01_Se_0.96_Te_0.04_ sample at 750 K. Furthermore, the thermoelectric performance of SPST was seriously deteriorated by formation of *n*-type SnSe_2_. If the interference of SnSe_2_ is removed, the thermoelectric performance of SPST will be greatly improved.

## Figures and Tables

**Table 1:** The relative density and density of all samples at room temperature.

Samples	Density (g cm^−3^)	The Relative Density
SnSe	5.88	95.15%
Sn_0.94_Pb_0.01_Se	6.00	96.93%
Sn_0.94_Pb_0.01_Se_0.99_Te_0.01_	5.93	95.80%
Sn_0.94_Pb_0.01_Se_0.98_Te_0.02_	6.04	97.58%
Sn_0.94_Pb_0.01_Se_0.97_Te_0.03_	5.99	96.77%
Sn_0.94_Pb_0.01_Se_0.96_Te_0.04_	5.97	96.45%
Sn_0.95_Se	6.04	97.73%

**Table 2:** Band gaps estimated by Seebeck coefficients for the SPST samples and pristine SnSe.

Samples	*E_g_* (eV)
SnSe	0.527
Sn_0.94_Pb_0.01_Se	0.401
Sn_0.94_Pb_0.01_Se_0.99_Te_0.01_	0.446
Sn_0.94_Pb_0.01_Se_0.98_Te_0.02_	0.466
Sn_0.94_Pb_0.01_Se_0.97_Te_0.03_	0.490
Sn_0.94_Pb_0.01_Se_0.96_Te_0.04_	0.503

**Table 3:** The numerical values of the various parameters for all samples at 750 K.

Samples	*σ*(Scm^−1^)	*S*(μVK^−1^)	*PF*(μWK^−2^ cm^−1^)	*κ*(Wm^−1^K^−1^)	*zT*
SnSe	22.46	377.73	3.20	0.43	0.55
Sn_0.94_Pb_0.01_Se	24.75	235.50	1.37	0.38	0.27
Sn_0.94_Pb_0.01_Se_0.99_Te_0.01_	22.56	250.03	1.41	0.35	0.306
Sn_0.94_Pb_0.01_Se_0.98_Te_0.02_	21.61	288.81	1.80	0.36	0.37
Sn_0.94_Pb_0.01_Se_0.97_Te_0.03_	21.58	314.29	2.13	0.31	0.52
Sn_0.94_Pb_0.01_Se_0.96_Te_0.04_	20.10	340.14	2.33	0.29	0.607
Sn_0.95_Se	25.84	306.68	2.31	0.29	0.60

## Data Availability

Data are contained within the article.
